# Organization of the Addax Major Histocompatibility Complex Provides Insights Into Ruminant Evolution

**DOI:** 10.3389/fimmu.2020.00260

**Published:** 2020-02-25

**Authors:** Chaokun Li, Rui Huang, Fangyuan Nie, Jiujie Li, Wen Zhu, Xiaoqian Shi, Yu Guo, Yan Chen, Shiyu Wang, Limeng Zhang, Longxin Chen, Runting Li, Xuefeng Liu, Changming Zheng, Chenglin Zhang, Runlin Z. Ma

**Affiliations:** ^1^School of Life Sciences, University of Chinese Academy of Sciences, Beijing, China; ^2^State Key Laboratory of Molecular Developmental Biology, Institute of Genetics and Developmental Biology, Chinese Academy of Sciences, Beijing, China; ^3^Molecular Biology Laboratory of Zhengzhou Normal University, Zhengzhou, China; ^4^Beijing Key Laboratory of Captive Wildlife Technologies, Beijing Zoo, Beijing, China

**Keywords:** MHC, *Addax nasomaculatus*, ruminant, chromosome inversion, evolution, *DY*

## Abstract

Ruminants are critical as prey in transferring solar energy fixed by plants into carnivorous species, yet the genetic signature of the driving forces leading to the evolutionary success of the huge number of ruminant species remains largely unknown. Here we report a complete DNA map of the major histocompatibility complex (MHC) of the addax (*Addax nasomaculatus*) genome by sequencing a total of 47 overlapping BAC clones previously mapped to cover the MHC region. The addax MHC is composed of 3,224,151 nucleotides, harboring a total of 150 coding genes, 50 tRNA genes, and 14 non-coding RNA genes. The organization of addax MHC was found to be highly conserved to those of sheep and cattle, highlighted by a large piece of chromosome inversion that divided the MHC class II into IIa and IIb subregions. It is now highly possible that all of the ruminant species in the family of Bovidae carry the same chromosome inversion in the MHC region, inherited from a common ancestor of ruminants. Phylogenetic analysis indicated that *DY*, a ruminant-specific gene located at the boundary of the inversion and highly expressed in dendritic cells, was possibly evolved from *DQ*, with an estimated divergence time ~140 million years ago. Homology modeling showed that the overall predicted structure of addax DY was similar to that of HLA-DQ2. However, the pocket properties of P1, P4, P6, and P9, which were critical for antigen binding in the addax DY, showed certain distinctive features. Structural analysis suggested that the populations of peptide antigens presented by addax DY and HLA-DQ2 were quite diverse, which in theory could serve to promote microbial regulation in the rumen by ruminant species, contributing to enhanced grass utilization ability. In summary, the results of our study helped to enhance our understanding of the MHC evolution and provided additional supportive evidence to our previous hypothesis that an ancient chromosome inversion in the MHC region of the last common ancestor of ruminants may have contributed to the evolutionary success of current ruminants on our planet.

## Introduction

The major histocompatibility complex (MHC) is the genetic foundation for mammals' ability to defend against microbial pathogens (1). Molecules encoded by MHC genes play pivotal roles in innate and adaptive immune responses (2). While MHC class I molecules bind with β_2_-microglobulin to present endogenous antigen peptides to CD8^+^ cytotoxic T lymphocytes (3), MHC class II molecules present exogenous antigen peptides from lysosomes and other endocytosis compartments to CD4^+^ T lymphocytes (4). The structures of MHC class I and class II proteins are very similar, with both types consisting of a membrane-distal peptide-binding domain and a membrane-proximal immunoglobulin domain (5). The presentation of antigens by MHC class II molecules relies on the structure of the peptide-binding groove (PBG), which is composed of two α-helices on the sides and an eight-strand β-sheet at the bottom (6). Four pocket structures, named P1, P4, P6, and P9, are critical for the MHC class II molecule to bind the correct antigen (7). P7 and P10 can also influence peptide-binding specificity (8).

The genomic organization of MHC in mammals is relatively conserved, with a basic orientation of class I, class III, and class II (9). However, distinct from other animals, the MHC class II region in sheep and cattle is composed of two discontinuous regions, class IIa and class IIb, which result from a substantial autosome inversion (10, 11). Recent studies have indicated that this chromosome inversion was also found in the Yangtze porpoise, killer whale, and seven other cetaceans (12, 13). However, the evolutionary significance of the inversion in the MHC class II region remains elusive.

The MHC region is characterized by high polymorphism and is rich in repetitive sequences (14, 15). The interspersed repeats are the major constituent of mammalian genomes, accounting for 40–50% of the total genome sequence (16–19). Most of these repeats are inactive, incomplete copies of transposable elements (TEs) (20), which are involved in remodeling the genomic structure and are proposed to be the main drivers of genome evolution (21). The interspersed repeats consist of retrotransposons and DNA transposons, which differ in their propagation in the genome (22). The retrotransposons can be further divided into long interspersed elements (LINEs), short interspersed elements (SINEs), and long terminal repeat elements (LTRs) (23). In mammalian genomes, four clades of LINE elements are well-known: retro-transposable element (RTE), L1, L2, and CR1 (chicken repeat 1)/L3 element (23). RTE elements, which are absent from human and rodent genomes, can transpose their lineage-specific SINE elements by encoding the transposable machinery in ruminants and marsupials (24). The L1 element is a prevalent interspersed repeat sub-type in mammalian genomes (21).

MHC genes are proposed to undergo the “birth-and-death” evolutionary process, in which new genes emerge from gene duplications, and some duplicated genes are retained in the genome for a long time, whereas others are inactivated or deleted due to the accumulation of deleterious mutations (25). The evolutionary rate of MHC class I genes is faster than that of MHC class II genes, making class II genes a better resource to construct phylogenetic trees that reflect the evolutionary history of MHC genes (26).

Ruminantia, which is a suborder of Artiodactyla, includes ~200 herbivores (27, 28). The most evident characteristic of ruminants is a four-chambered stomach, which provides the physical foundation for converting lignocellulose-rich plant materials into animal protein (19). The rumen emerged ~35–40 million years ago (MYA) (29), which coincided with the emergence of grass. The rumen is a large microbial ecosystem, consisting of bacteria, protozoa, archaea, fungi, and viruses (30). These microorganisms and ruminants have mutually beneficial symbiosis. MHC molecules play important roles in the crosstalk between rumen microorganisms and the host immune system (31). However, the immune homeostasis of ruminants is also challenged by these microorganisms, and the mechanism underlying this homeostasis remains unclear.

Addax (*Addax nasomaculatus*) belongs to the subfamily Hippotraginae, family Bovidae. It is a critically endangered species listed on the IUCN red list (32). The addax is phylogenetically most distant from sheep (subfamily Caprinae) and cattle (subfamily Bovinae) in the family Bovidae (27). Recent studies indicate that due to genetic drift and population fluctuation, small populations are prone to losing functional MHC alleles (33). Therefore, a thorough understanding of the addax MHC region will provide important theoretical guidance for the protection of this endangered species and insights into the evolutionary history of the MHC region in the family Bovidae.

In this study, we focused on the comparative organization of the ruminant MHC by generating a detailed sequence map of the MHC region in the addax genome. A total of 47 overlapping BAC clones, previously mapped to the addax MHC region, were sequenced and annotated in detail, providing the basis for a detailed comparative analysis of the MHC regions of the addax and other mammalian species.

## Methods

### BAC Sequencing, Assembly, and Gene Annotation

Positive BAC clones covering the addax MHC region were selected according to the previously published paper (34). A DNA source was acquired from a male addax in Beijing Zoo. BAC plasmid DNA was extracted using a QIAGEN Large-Construct kit (Qiagen, Hilden, Germany) to avoid *E. coli* genomic DNA contamination. BAC plasmids were sequenced on a HiSeq 2000 platform (Illumina, San Diego, CA, USA) by Shanghai Majorbio Bio-Pharm Technology Company (Shanghai, China). After removing adapter sequences, poly-N reads (containing >10% N), and low-quality reads, the resulting clean reads were used to assemble the sequence using SOAP v2.04 (35). Gaps were closed by the primer-walking method during the assembly process. Meanwhile, GapCloser v1.12 software was used to fill in gaps and perform base corrections in the local assembly. Two complete sequences covering the addax MHC region were submitted to the GenBank database, with accession numbers MN128535-MN128536. Genes were predicted using Fgenesh (36), and the predicted protein sequences were aligned with the NR, STRING, and GO databases using BLAST (37) to acquire the annotation information. Non-coding genes/components were predicted by Rfam database analysis tools (38).

### Repetitive Element Detection and Comparative Analysis of MHC Regions

Interspersed repetitive elements in the addax MHC region and several other representative mammalian species were characterized to obtain the complete picture of the distribution of these elements in mammalian MHC regions. Repetitive elements were analyzed by the RepeatMasker Web Server (39). The DNA source was set as artiodactyls and whales when analyzing the addax MHC sequences. Homologous MHC region sequences and annotation data from sheep (*Ovis aries*), goat (*Capra hircus*), cattle (*Bos taurus*), water buffalo (*Bubalus bubalis*), red deer (*Cervus elaphus*), sperm whale (*Physeter catodon*), pig (*Sus scrofa*), horse (*Equus caballus*), and human (*Homo sapiens*) were downloaded from Ensemble and NCBI databases for comparative analysis. Different types of elements were identified according to the results of RepeatMasker (Table S1). After the masked sequences were acquired from RepeatMasker, Pipmaker (40) was used to perform a self-dot matrix analysis. MHC sequences from the addax, sheep, cattle, red deer, sperm whale, pig, and human were analyzed by VISTA plot (41) to compare the architectural conservation of the mammalian MHC region.

### Phylogenetic Analysis and Estimation of the Divergence Time of the MHC Genes

The PBG was encoded by exon 2 in the MHC class II genes, which was influenced by greater selective pressure from pathogens than other exons (42). To exclude the selective pressures from pathogens and reflect the true evolutionary process of the MHC class II genes, the sequences from exon 3 and exon 4 of *DO, DR, DY*, and *DQ* genes were used to construct the phylogenetic tree of the MHC class II A and B genes, respectively. *DP* genes were not included because they were only found in two of the species included in the analysis (human and pig). *DM* genes were excluded due to undertaking different functions from *DO, DR, DY*, and *DQ* genes in the process of antigen presentation (4). Sequences were aligned using MUSCLE in the codon alignment mode. The “find the best DNA/protein model” function in MEGA X (43) was used to obtain the best model for the construction of the phylogenetic tree. MEGA X was also used to construct a maximum likelihood phylogenetic tree and a neighbor-joining tree. MHC class II genes from cattle (*Bos taurus*), water buffalo (*Bubalis bubalis*), sheep (*Ovis aries*), goat (*Capra hircus*), addax (*Addax nasomaculatus*), Tibetan antelope (*Pantholops hodgsonii*), red deer (*Cervus elaphus*), forest musk deer (*Moschus berezovskii*), sperm whale (*Physeter catodon*), minke whale (*Balaenoptera acutorostrata*), bottlenose dolphin (*Tursiops truncates*), pig (*Sus scrofa*), Bactrian camel (*Camelus bactrianus*), and human (*Homo sapiens*) were used to construct the phylogenetic tree. The sequences of exon 3 and exon 4 of the zebrafish *A1* and *DAB1* genes were used as outgroups for the MHC class II A and B genes, respectively. The accession numbers of the genes used in the construction of the phylogenetic tree are shown in Table S2. Sequences of the MHC class II genes of the forest musk deer (*Moschus berezovskii*) were acquired by a local BLAST search of the sequencing data reported by Fan et al. (44). The divergence time of the MHC genes was estimated using BEAST (45), with a relaxed molecular clock. Calibration times were acquired from the fossil records of humans and other mammals (94.6–106.4 MYA), camels and pigs (57.2–65.7 MYA), and sheep and cattle (17.8–24.5 MYA) (19).

### Homology Modeling of *DY*

Addax DYA and DYB amino acid sequences were translated from their gene coding sequences and then submitted to SWISS-MODEL to perform homology modeling (46). The HLA-DQ2 complex with PDB ID of 4D8P was chosen as the template for model construction. PYMOL (https://pymol.org/2/) software was used to present and compare the structure of DY and DQ molecules. Root mean square deviations (RMSD) were calculated by aligning the predicted structure of addax DY against the structure of HLA-DQ2 by using PYMOL.

## Results

### Gene Annotation and Organization of Addax MHC

**Figure 1:**
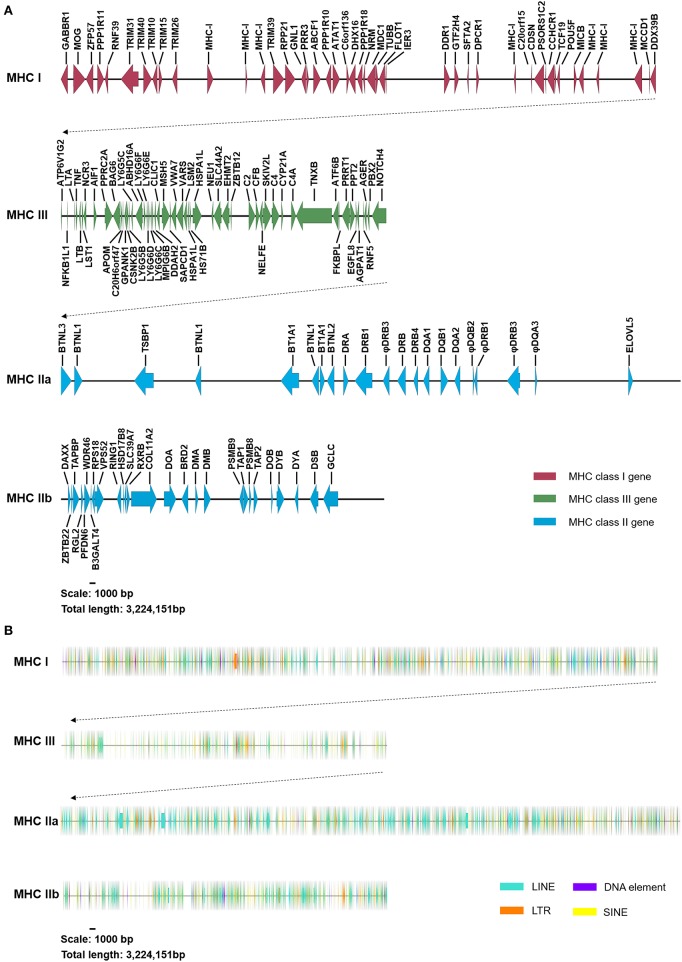
Genomic structure and content of the addax MHC region. **(A)** The addax MHC region was segregated into two subregions (MHC class I-III-IIa and class IIb). Genes in the addax MHC class I, III, and II regions are indicated by red, green, and blue arrows, respectively. The length of the addax MHC region is 3,224,151 nucleotides, harboring a total of 150 coding genes. Detailed gene annotations and their parameters are presented in Table S3. **(B)** Four types of repetitive elements, LINEs, LTRs, DNA elements, and SINEs, are indicated by green, orange, purple, and yellow arrows, respectively. The detailed distribution of repetitive elements can be seen in Figure S1.

The DNA sequencing of 47 overlapping BAC clones generated a high-accuracy DNA sequencing map in the addax MHC, with a detailed gene annotation (Figure 1, Figure S1). The number of nucleotides for BAC contig I and II, covering addax MHC class I-III-IIa and IIb, were 2,669,394 and 554,757 bp, respectively. A total of 150 new genes and 50 tRNA genes were annotated in the addax MHC region (Tables S3, S4). Fourteen non-coding RNA genes were also predicted using the Rfam database (Table 1). The genomic organization in the addax (subfamily Hippotraginae) MHC region was highly conserved with those of sheep (subfamily Caprinae) and cattle (subfamily Bovinae) (Figure S2). Their MHC class II regions were all interrupted by a large piece of chromosome inversion into class IIa and class IIb sub-regions (Figure S2). Based on the fact that three subfamilies so far examined in the Bovidae all contained the said chromosome inversion, it is possible that all of the species in the ruminant family of Bovidae carry the same piece of chromosome inversion in their MHC regions.

**Table 1:** List of non-coding RNA genes identified in the MHC region by Rfam analysis.

**Gene name**	**Rfam accession no**.	**Start**	**End**	**Bits score**	***E*-value**	**Strand**	**Contig**
5S_rRNA	RF00001	9479	9373	56.2	1e^−09^	-	1
sno_ZL8	RF02725	1402684	1402603	75.6	1.5e^−16^	-	1
SNORD52	RF00276	1403091	1403025	86.1	1.1e^−18^	-	1
SNORD48	RF00282	1403728	1403666	89.6	4.1e^−22^	-	1
SNORA38	RF00428	1568339	1568209	163	6e^−36^	-	1
SNORD83	RF00137	1647604	1647682	95.3	4.3e^−20^	+	1
SNORD83	RF00137	1649843	1649912	36.7	0.00018	+	1
SNORD83	RF00137	1652452	1652528	70.7	1.5e^−13^	+	1
U6	RF00026	1970956	1971059	86.9	1.4e^−20^	+	1
U6	RF00026	2056441	2056547	102.8	3.6e^−25^	+	1
mir-877	RF00912	2226947	2226863	96.3	2.4e^−20^	-	1
7SK	RF00100	2335305	2335631	171	1.4e^−46^	+	1
ZNRD1-AS1_2	RF02219	2607946	2607871	95.2	9e^−23^	-	1
mir-219	RF00251	449881	449952	89.3	6.9e^−21^	+	2

We noticed that the *DXO* (decapping exoribonuclease) locus that was found in the MHC class III region of sheep and cattle was not detected at the corresponding location in the addax (Figure S2). As the existence of the addax *DXO* gene has been confirmed by PCR and sequencing (data not shown), it is highly possible that the gene may have been rearranged to other genomic regions.

### Comparison of the TE Landscape Among Representative Mammalian Species

**Figure 2:**
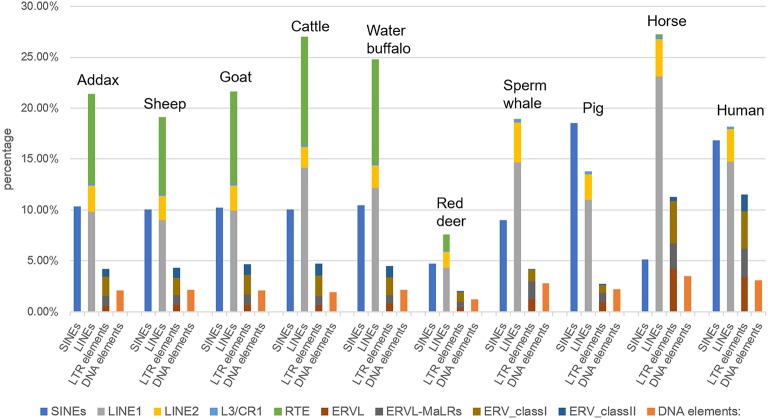
Frequency of four types of repetitive elements (SINEs, LINEs, LTR elements, and DNA elements) in the MHC region of representative mammalian species. The MHC region sequences of sheep, goat, cattle, water buffalo, sperm whales, pig, horse, and human were downloaded from the NCBI Genome (https://www.ncbi.nlm.nih.gov/genome/) and Ensembl (http://asia.ensembl.org/index.html) databases. LINEs were further classified into LINE1, LINE2, L3/CR1, and RTE elements.

Our results showed that the constitution of the TE landscape in the addax MHC was highly similar to those of sheep and goat (Figure 2). The addax MHC region consisted of 38.16% interspersed repeats, which was a similar frequency to that of sheep (35.69%) and goat (38.77%), which in turn were low compared to cattle (43.75%) and water buffalo (41.94%) (Table S1). In general, the percentage of the four main classes of repetitive elements (SINEs, LINEs, LTR elements, and DNA elements) in the addax MHC region was conserved with the sheep and goat MHC regions in the subfamily Caprinae (Table S1).

As an exception, the TE element in the MHC of red deer consisted of 15.71% of the MHC (47), which was a much lower frequency than in other mammalian species (Table S1), although the repeat elements make up 22.73% of the entire red deer genome (47). The ratio of RTE elements to the total interspersed repeats showed little fluctuation between members of Bovidae, ranging from 21.1% in the MHC region of sheep to 24.4% in that of water buffalo (Table S1).

LINE was found to be the most abundant repeat element in both the MHC region and the entire genome of the mammals listed above, except for pig (Figure 2). In these ten representative mammalian species, the pig had the most abundant SINEs in its MHC region, at 18.54%, such that SINEs exceeded LINEs as the most abundant interspersed repeat elements. This may have been due to the centromere, a specialized chromosomal region with a highly repetitive sequence, located in the middle of class III and class II genes in the porcine MHC region (48). In addition, we observed that the high frequency of LINEs in the genome of the sperm whale (85.38% of all repeats) (49) was not congruent with their frequency in the MHC region (53.91% of all repeats) (Table S1). This may be a result of the contraction of MHC region genes that were specific to the sperm whale (49).

The detailed distribution of repetitive elements in the addax MHC region was determined by dot-plot analysis. This entire addax MHC region was divided into MHC class I, class IIa, class IIb, and class III to improve the resolution. In the addax MHC class I region, direct repeats localized from φ*IFIM3* to *MICB* (Figure S3A). This direct repeat pattern was also detected in the corresponding regions of the sheep and cattle genome but not in the pig genome (Figure S3A), which indicated that this may be a common feature shared by Bovidae. In the addax MHC class IIa region, the direct repeat elements mainly localized to the φ*DQA3*-*DRB1* region (Figure S3B), which mostly consisted of pseudogenes. Direct repeats were also found in the homologous region of cattle, pig, and human genomes (Figure S3B), suggesting that this repeat pattern is a common feature in mammalian MHC class IIa regions. Interestingly, the tandem repeat in the addax MHC class III region was also observed in the corresponding region of cattle, pig, and human (Figure S3C). We found that this pattern was due to tandem repeats in the *TNXB* gene. No direct or inverted repeats were found in the MHC class IIb region of the addax (Figure S4).

### Comparative Analysis of Mammalian MHC Regions

**Figure 3:**
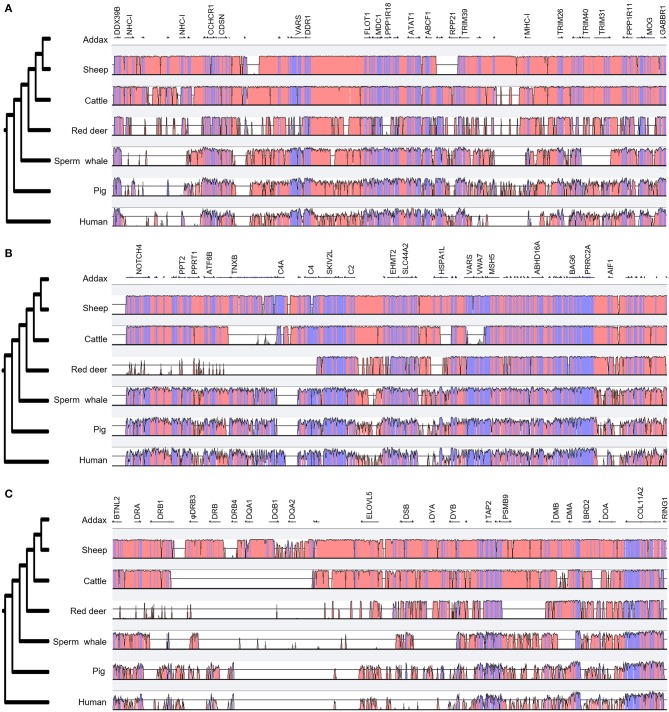
Comparison of the genomic organization of three MHC regions of seven mammalian species. A comparison of MHC class I, class III, and class II regions in the addax, sheep, cattle, red deer, sperm whale, pig, and human are presented in **(A–C)**, respectively. The phylogenetic trees listed on the left were generated by the VISTA program. MHC class IIb regions of the addax, sheep, cattle, and red deer were inverted for comparative analysis with other species. Coding and non-coding regions are depicted as light blue and light pink, respectively.

VISTA plot results showed that the MHC region of the sheep was most similar in structure and content to the addax (Figure 3). The major differences between the MHC regions of cattle and the addax were located in the *TNXB, HSPA1A*, and *VARS* regions (Figure 3B). The MHC class III region was the most conserved among three MHC regions (Figure 3B). This may be attributed to the fact that genes localized to the MHC class III region encoded proteins involved in the complement system and heat-shock proteins, which are highly conserved in the mammalian genome (2). The class II region was the least conserved, especially in the *DR*-*DY* region (Figure 3C).

### Phylogenetic Analysis of Mammalian MHC Class II Genes Showed That *DY* Genes Were Close to *DQ* Genes

**Figure 4:**
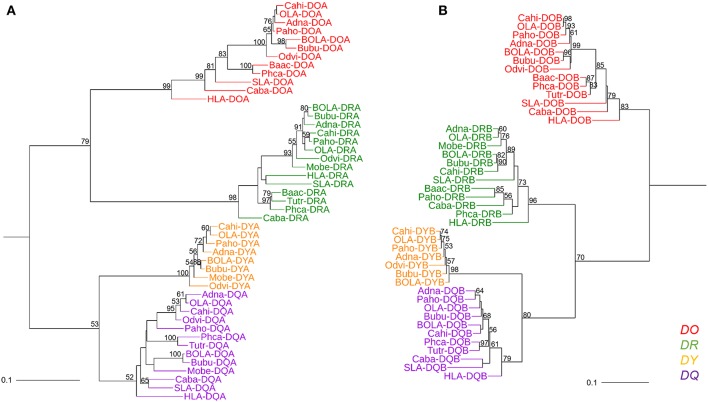
Phylogenetic tree of the MHC class II genes of fourteen mammalian species. **(A)** Phylogenetic tree of MHC class II A genes. **(B)** Phylogenetic tree of MHC class II B genes. These phylogenetic trees were constructed using the maximum likelihood method, based on the Kimura 2-parameter model. One thousand bootstrap replications were performed, and the percentages larger than 50 are shown next to the branches. The MHC class II genes of mammalian species are indicated by the combination of abbreviated Latin animal names and gene names. *DO, DR, DY*, and *DQ* genes are indicated by red, green, orange, and purple, respectively. The topology of the phylogenetic tree based on the maximum likelihood method and the neighbor-joining method was the same, but only the maximum likelihood tree is shown here.

Genes from various mammalian species formed separate groups of the *DR, DO, DQ*, and *DY*. The phylogenetic tree based on MHC class II A genes was not congruent with the tree based on class II B genes in terms of the topology of *DR* with the other gene clusters. In the phylogenetic tree of class II A genes (Figure 4A), *DRA* genes clustered with *DOA* genes, while *DYA* genes clustered with *DQA* genes. However, in the phylogenetic tree of class II B genes (Figure 4B), *DRB* genes first clustered with *DYB*-*DQB* genes and then with *DOB* genes. The phylogenetic trees of both class II A genes and class II B genes demonstrated that the relationship between *DY* genes and *DQ* genes was closer than the relationship between the other two class II genes (Figure 4).

**Figure 5:**
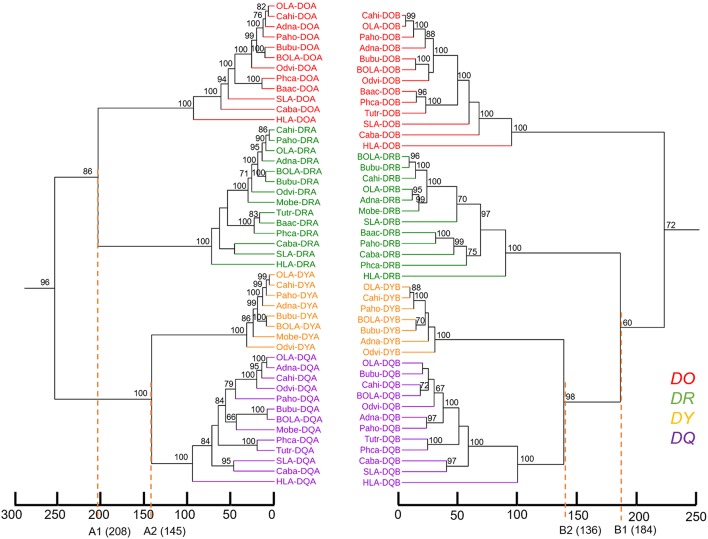
Estimation of divergence time of mammalian MHC class II genes. Phylogenetic trees were drawn, with branch lengths reflecting BEAST divergence age estimations of MHC class II A and B genes. Scale = millions of years before the present. The percentage of bootstrap values larger than 60 is shown next to the branches. The MHC class II genes of mammalian species are indicated by the combination of abbreviated Latin animal names and gene names. *DO, DR, DQ*, and *DY* genes are indicated by red, green, orange, and purple, respectively.

The divergence time of *DY* genes and *DQ* genes was ~140 million years ago (Figure 5). The divergence time of *DOA* and *DRA* was found to be ~208 MYA (Figure 5, A1 point), and that of *DYA* and *DQA* was ~145 MYA (Figure 5, A2 point). In contrast, the divergence time of *DYB* and *DQB* was ~136 MYA (Figure 5, B2 point), and that of *DRB* and *DYB*-*DQB* was ~184 MYA (Figure 5, B1 point).

### Comparison of *DY* and HLA-DQ2 Structure Revealed Distinctive Characteristics of the *DY* Antigen-Binding Pocket

**Figure 6:**
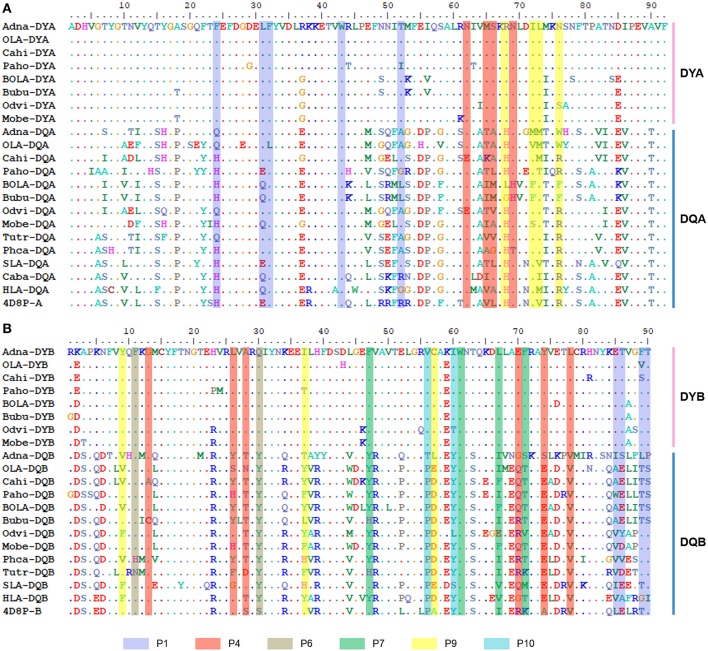
Alignment of key amino acids involved in antigen peptide binding of the α and β chains of DY and DQ from mammalian species. **(A)** Alignment of the amino acid sequences of the PBGs of DQA and DYA. **(B)** Alignment of the amino acid sequences of the PBG of DQB and DYB. Amino acids in different pockets that participated in antigen binding are shaded by different colors, as indicated above. The sequence names of DY and DQ from different mammalian species are indicated by the combination of abbreviated Latin animal names and gene names, except that 4D8P (PDB ID of HLA-DQ2) indicates the sequences of HLA-DQ2.

The alignment of amino acids containing P1–P10 pockets revealed that the sequence containing the PBG was more conserved in DY than in DQ among several mammalian species (Figures 6A,B). This was true even at α44–α54 and β84–β90, two clusters in the α and β chains of HLA-DQ2 containing extensive polymorphism (50, 51). Key amino acids at P1, P4, P6, and P9 sites in the DY α chain and β chain showed greater conservation than the corresponding sites in DQ (Figure 6). Only six amino acids (α32F and α43W in P1, α62N, and α69N in P6, β74A in P4, and β61W in P7) were conserved between DY and DQ. In particular, β78 in P4 and β56 in P10 were conserved in DYB and DQB, respectively. Therefore, the topology and characteristics of the PBG formed by DY may be quite different from that formed by DQ.

**Figure 7:**
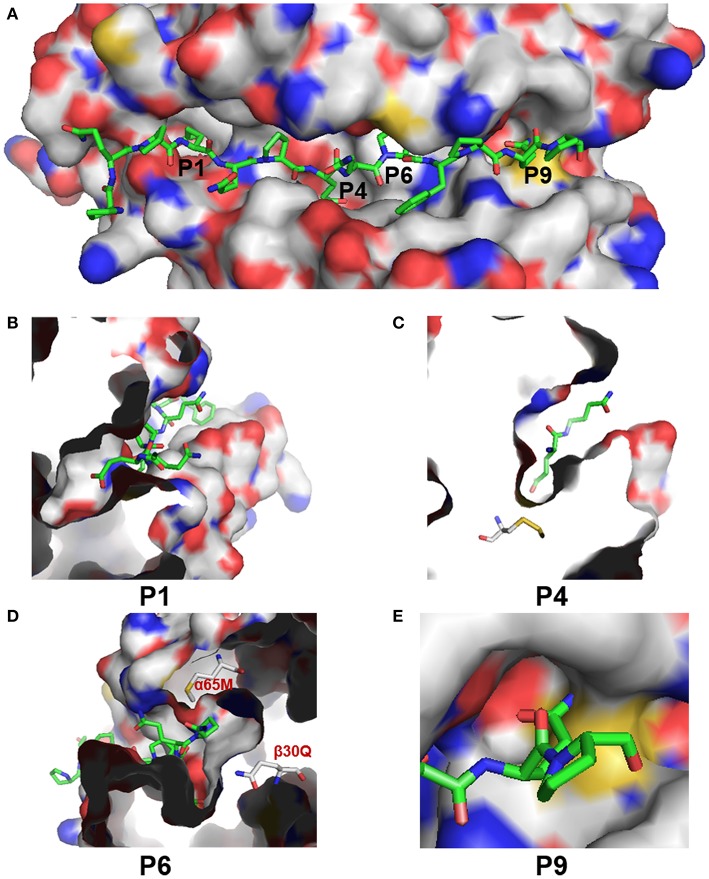
Structural characteristics of the PBG of addax DY. **(A)** Surface presentation of the PBG in addax DY with P1, P4, P6, and P9 indicated at the corresponding sites. C and H atoms are colored as gray. N, O, and S atoms are colored blue, red, and yellow, respectively. Antigen peptides (from HLA-DQ2) are shown as sticks, with carbon atoms colored green. **(B–E)** Zoomed-in view of the P1, P4, P6, and P9 pockets, respectively, in the PBG of addax DY.

The low RMSD value (0.086) indicated the high confidence of the predicted structure of addax DY. The overall predicted structure of DY resembled the structure of HLA-DQ2 (Figure S5A). Both proteins shared the canonical structure of the MHC class II PBG, formed by two α-helices at the sides and β sheets at the bottom (Figures S5B,C). However, there were some slight differences in their peptide-binding groove resulting from the different amino acids from β26 to β30 (Figure S6). We then focused on the predicted structures of the P1, P4, P6, and P9 pockets (Figure 7A), which confer the specificity of the anchor residues. These pockets can accommodate the side chain of the antigen peptide (7). A zoomed side view of P1, P4, and P6 from the side is shown in Figures 7B–D.

The differences in the P1 pocket were mainly electrostatic. For example, the change from residue His-α24 (in HLA-DQ6.2) to Phe-α24 (in DY) and the change from Arg-α52 (in HLA-DQ6.2) to Tyr-α52 (in DY) reduced the positive charge and may be expected to subtly modulate anchor specificity. Residue 66 in the α-chain (66α), which is part of the P4 pocket, changed from leucine to serine, increasing the hydrophilicity of the P4 pocket. Besides, the change from His-β70 (in HLA-DQ6.2) to Glu-β70 (in DY) introduced a negative charge in the P4 pocket. However, the most marked difference was in the P6 pocket. The predicted DY structure showed a distinctive P6 pocket, with a negative charge due to the presence of Gln-β30. Additionally, the presence of glutamine at 30β, along with methionine at α56, at the entrance of the P6 pocket in DY, sterically hindered the glutamate side chain to take on an appropriate conformation as it took in HLA-DQ2 (Figure 7D). In most MHC class II molecules, Asp-β57 forms a hydrogen bond with the peptide backbone and a salt bridge, within the P9 pocket, with Arg76α (7). However, Cys57β in DY sterically hindered the binding of the side chain of the antigen peptide and decreased the volume of the P9 pocket (Figure 7E). In addition, the change from Arg-α76 (in HLA-DQ6.2) to Asn-α76 (in DY) reversed the charge characteristics of the P9 pocket. These results showed that DY preferred to bind positively charged anchor residues at the P1, P4, P6, and P9 positions. These pocket characteristics (such as volume, hydrophobicity, and electrostatic charge) made DY quite different from HLA-DQ2 at the PBG, indicating that their presented antigen peptides were quite diverse.

## Discussion

In this study, we provided a detailed sequence map of the MHC region in the addax. This study may serve as a foundation for the estimation of genetic diversity in the addax MHC region, and the results may be used to avoid immune deficiency caused by inbreeding (52, 53), which would facilitate research aimed at the protection of the addax. Furthermore, a comparative analysis of the MHC region in several representative mammalian species and an analysis of the evolutionary history of mammalian MHC class II genes were performed to gain insights into ruminant evolution.

### The Low Level of Repetitive Elements in the MHC Region Is Not a Common Feature of the Subfamily Cervidae

Repetitive elements made up 22.73% of the red deer genome based on the genomic sequence of the red deer previously acquired by sequencing on the Illumina HiSeq 2000 platform, with a 74-fold coverage (47). The percentage of interspersed repeats in the genome of milu (*Elaphurus davidianus*) and hog deer (*Axis porcinus*), which were both in the subfamily of Cervinae of the family Cervidae, like red deer (*Cervus elaphus*), have been reported to be 41.04 and 38.9%, respectively (54, 55). It has been reported that interspersed elements made up only 10.9% of the white-tailed deer genome, which was too low to be plausible compared with other ruminants (56). The author speculated that this was attributable to the low genomic coverage during genome sequencing. Additionally, the interspersed repeat content in reindeer (*Rangifer tarandus*), which belongs to the subfamily Odocoileinae of the family Cervidae, like white-tailed deer (*Odocoileus virginianus*), was 35.71% (57). Therefore, this low percentage of interspersed repeats is likely to be a characteristic of red deer rather than a characteristic of the subfamily Cervinae. However, additional genomic information from Cervidae species is needed to confirm this.

### Additional Evidence for the Hypothesis of Ancestral Chromosome Rearrangement in Ruminants

**Figure 8:**
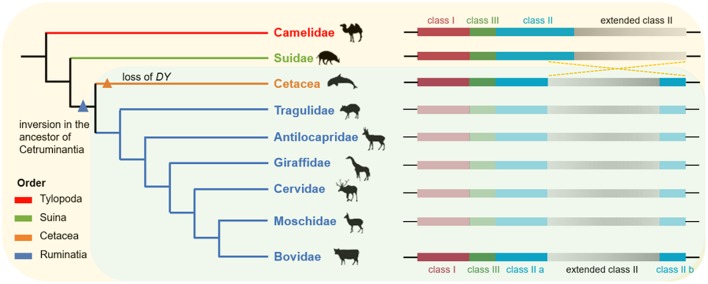
The hypothesis of ancient chromosome inversion in the ancestor of Cetruminantia. Genomic organization in the MHC region of Artiodactyla was compared with their phylogenetic relationships, listed on the left. This phylogenetic tree was adapted from Chen et al. (28). The organization of the MHC region in Suidae and Camelidae was inferred from the chromosome level genome assembly of pig and camel from the NCBI Genome database. This ancient inversion in the MHC region of the other five Ruminantia families remained to be determined due to the lack of genome information or poor genome assemblies.

This hypothesis of ancestral chromosome rearrangement in ruminants has been supported by previous studies comparing the MHC sequences of cattle and sheep with those of non-ruminants (such as humans, chimpanzees, and mice) (11, 58). Considering that subfamily Hippotraginae is phylogenetically most distant from subfamily Caprinae Bovinae in the family Bovidae (Figure S7), our results proposed that, at least in the Bovidae, an inversion in the MHC class II region divided the MHC class II region into two discontinuous subregions. In addition, some studies have reported that the MHC class II region of the Yangtze finless porpoise, killer whale, and seven other cetaceans were also divided into two separate sub-regions (12, 13). Taken together, these findings suggested that the inversion of the MHC class II region may be a common feature shared by cetaceans and ruminants (Figure 8). The inversion of the MHC class II region was not found in camels (suborder Tylopoda) or domestic pigs (suborder Suina), suggesting that this inversion occurred after the divergence of Suina and Cetartiodactyla but before the divergence of Cetacea and Ruminantia. As the most important mechanism of speciation, chromosome inversion may have contributed to the emergence of reproductive barriers and made taxa less likely to collapse following secondary contact (59, 60). Therefore, we propose that this inversion in the MHC class II region of cetaceans and ruminants may have facilitated the independent evolution of cetaceans and ruminants.

### *DY* Might Evolve From the Duplication of *DQ*

There have been few studies on *DY*, which appeared to be a functionally constrained ruminant gene (61, 62). *DY* is closest to *DQ* on the phylogenetic tree of the MHC class II genes (Figure 4). Besides, if inversion fragments in the ruminant MHC class II region were reversed, the location of the *DY* gene would be next to the *DQ* gene. Therefore, our finding provided new evidence for the hypothesis that *DY* may have originated from the duplication of *DQ* (47). The *DYB* pseudogene has been reported in the MHC region of domestic pigs and giant pandas (order Carnivora) (48, 63). This suggested that the *DY* gene may have emerged before the divergent evolution of the orders Artiodactyla and Carnivora. This was consistent with the divergence time of *DY* and *DQ* genes estimated in this study, which was ~140 MYA (Figure 6). This was even earlier than the divergence time of the superorder Laurasiatheria (including Carnivora, Perissodactyla, and Artiodactyla) and the superorder Euarchontoglires (94–100 million years ago) (63).

To date, the inversion of the MHC class II region has only been found in ruminants and cetaceans, whereas *DY* genes (including pseudogenes) have been detected in Ruminantia, Suina, and Carnivora (48, 61–63). Therefore, we propose that the *DY* genes emerged earlier than the inversion in the MHC class II region. However, *DY* genes were only retained in the ruminant genome, thus shaping the current genomic structure of the mammalian MHC region.

### *DY* Might Contribute to Microbial Regulation and Thus Improve the Environmental Suitability of Ruminants

There are numerous complex reactions between rumen microorganisms, their products, and the host immune system (31). Rumen homeostasis disorders may cause systemic inflammation in Holstein bulls and goats (64, 65). The balance between immune tolerance and the inflammatory response is regulated by communication between innate immune cells and microorganisms, in which dendritic cells play an important role (66, 67). Studies have shown that DY in the dendritic cells of ruminants may play a role in regulating the balance between immune tolerance and the inflammatory response of dendritic cells (61). Therefore, DY may have facilitated the adaption of ruminants to their environment and the improvement of the environmental suitability of ruminants in the evolutionary process. Cetaceans have the same terrestrial herbivorous ancestor as ruminants from before they adopted a dietary switch from being herbivores to carnivores, as they re-entered the oceans ~50 million years ago (68, 69). Although they retained the same multi-chambered stomach structure, their gut microbe diversity is significantly lower than in ruminants (68, 70). We propose that the loss of DY in cetaceans might be associated with this dietary switch and the habitat transition from land to water.

In this study, we first compared the protein structure of two MHC class II molecules, DY and DQ. We then established a preliminary association between rumen immunity and the MHC region and provided a hypothetical explanation that an ancient chromosome inversion occurred in the MHC region in the last common ancestor of ruminants, which serves as a signature of their evolutionary success.

Overall, the observed correlation between the MHC re-arrangement via the ancestral chromosome inversion and the evolutionary prevalence of ruminant species are preliminary in terms of solid experimental evidence. Rather, our studies helped to reveal the prevalence of this inversion in ruminants' MHC region. The detailed antigen-presenting mechanism of DY needs to be demonstrated in dendritic cells. In the future, the construction of a *DY* gene-knockout animal model and systematic evaluation of the rumen immune hemostasis of KO animals would help to provide a better understanding of DY function in relation to ruminant speciation.

## Data Availability Statement

The datasets generated for this study can be found in the NCBI Genbank database with accession numbers MN128535—MN128536.

## Author Contributions

CL and RM designed the experiments. CL, RH, and FN carried out data analysis. JL, WZ, XS, YG, YC, and SW collected data. LC, LZ, and RL contributed to the discussion. XL, CZhe, and CZha collected samples. RM and CL wrote the manuscript. RM supervised the studies.

### Conflict of Interest

The authors declare that the research was conducted in the absence of any commercial or financial relationships that could be construed as a potential conflict of interest.
