# Supplementary material for: Organization of the Addax Major Histocompatibility Complex Provides Insights Into Ruminant Evolution
Source: Front Immunol. 2020 Feb 25;11:260. doi: 10.3389/fimmu.2020.00260 (PMC7053375; doi:10.3389/fimmu.2020.00260)
Supplement: Supplementary file 1 [file Data_Sheet_1.docx]

**Supplementary material**

**
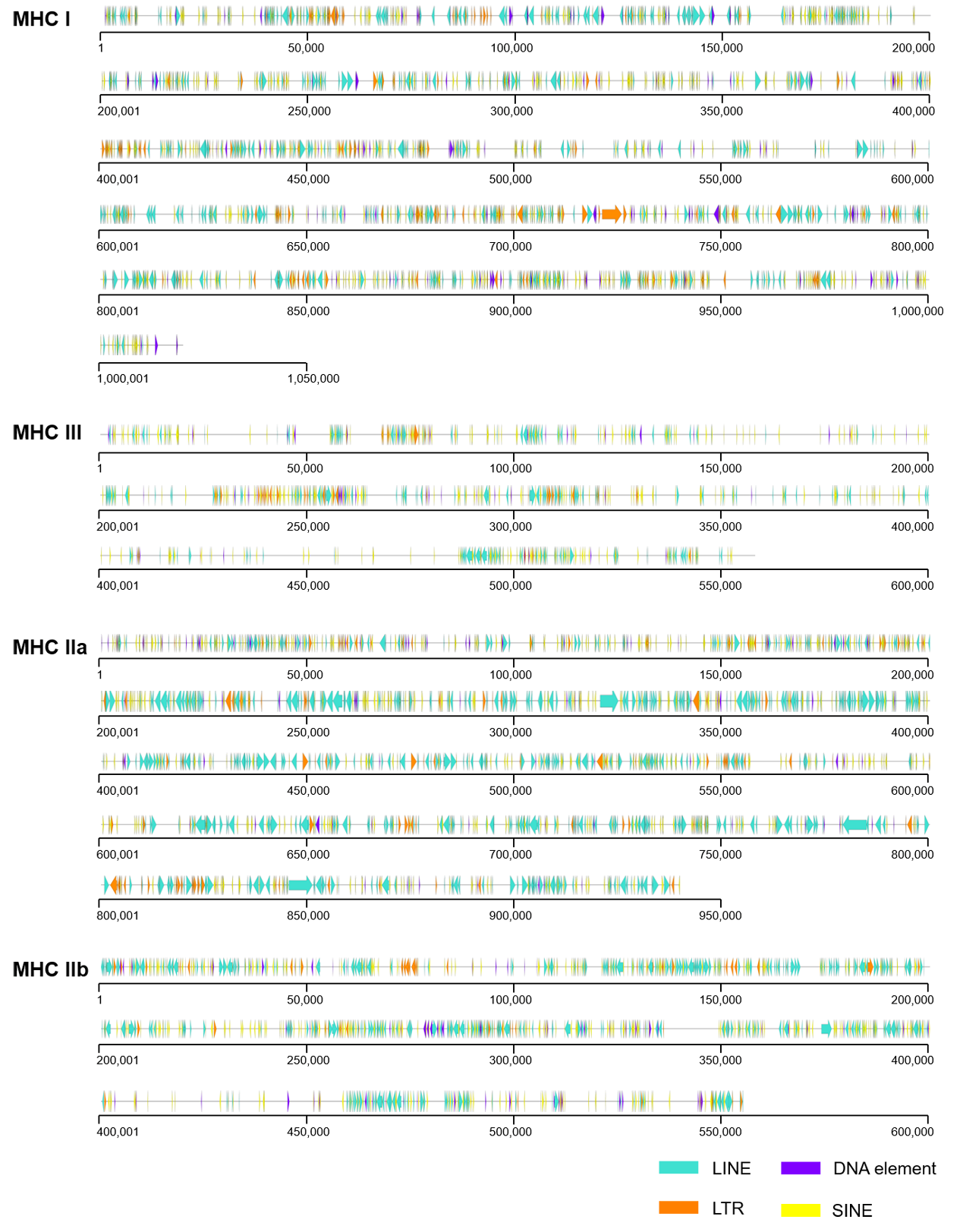
**

**Figure S1 | The distribution of repetitive elements in the addax MHC region.**


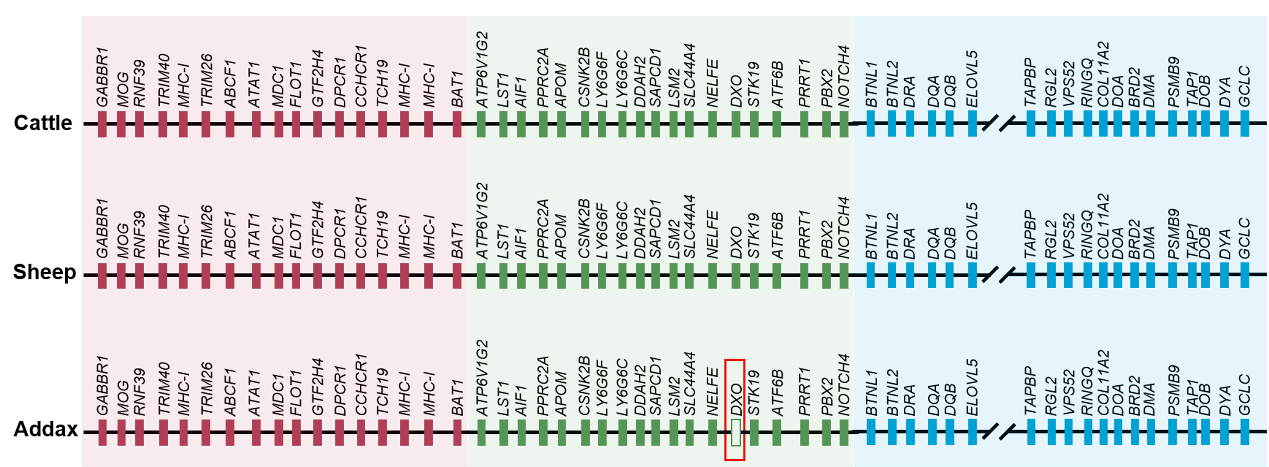


**Figure S2** | **Comparison of gene organization in** **addax, sheep, and cattle in the MHC region (not all annotated genes are shown here).** Red, green, and blue boxes indicate MHC class I, class III, and class II genes, respectively. The hollow box represents the *DXO* gene deleted in the class III region of the addax. The MHC class II regions in these three species are separated by a piece of non-MHC chromosome inversion (indicated by two short slashes).

**
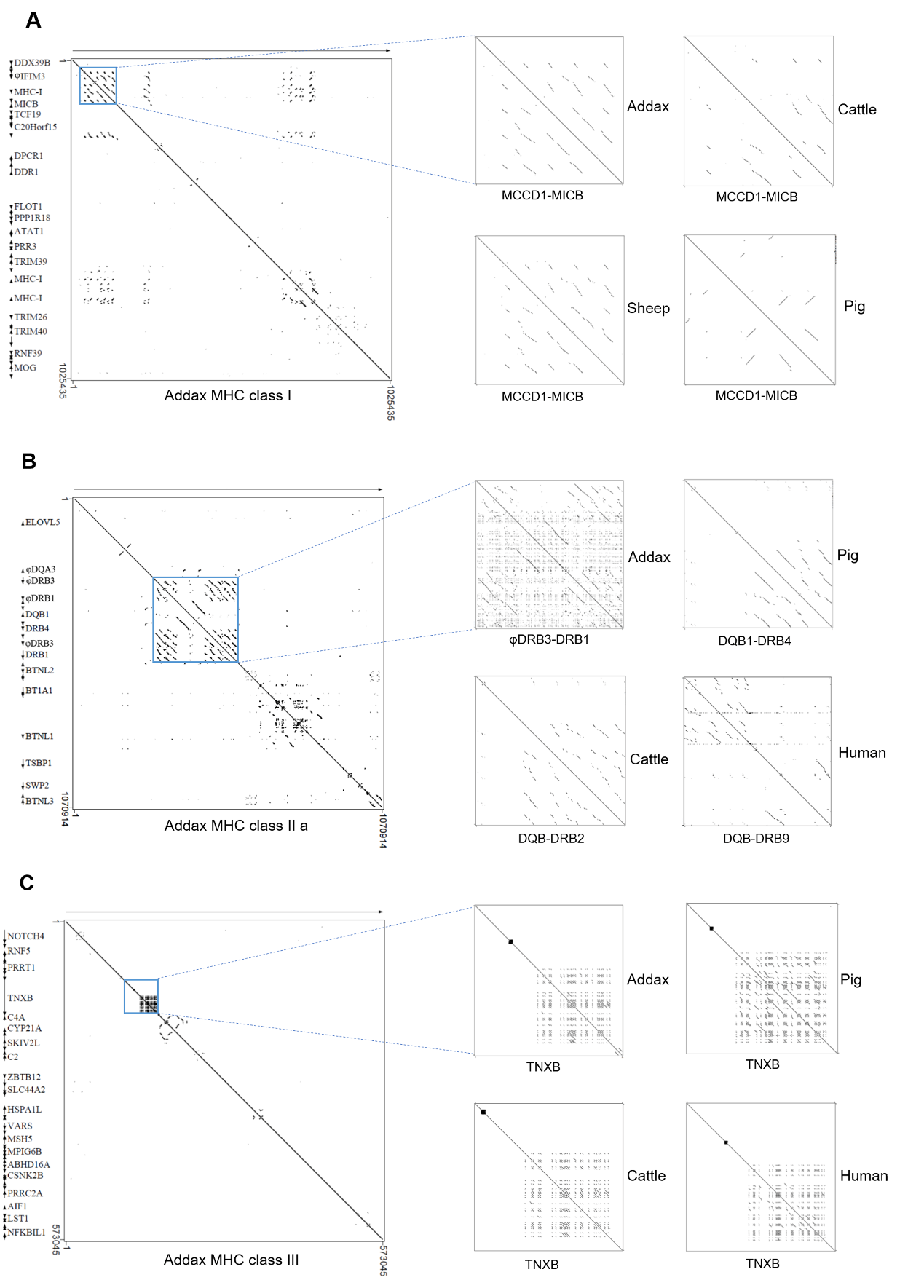
Figure S3** | **Dot plot analysis of the addax MHC genomic region.** The self-dot plot analysis of the addax MHC class I, IIa, and III are shown in **A** to **C**, respectively. Zoomed-in view of the corresponding region in sheep, cattle, pig, and human are shown on the right. Genes are listed on the y-axis, with their orientations indicated by arrows.


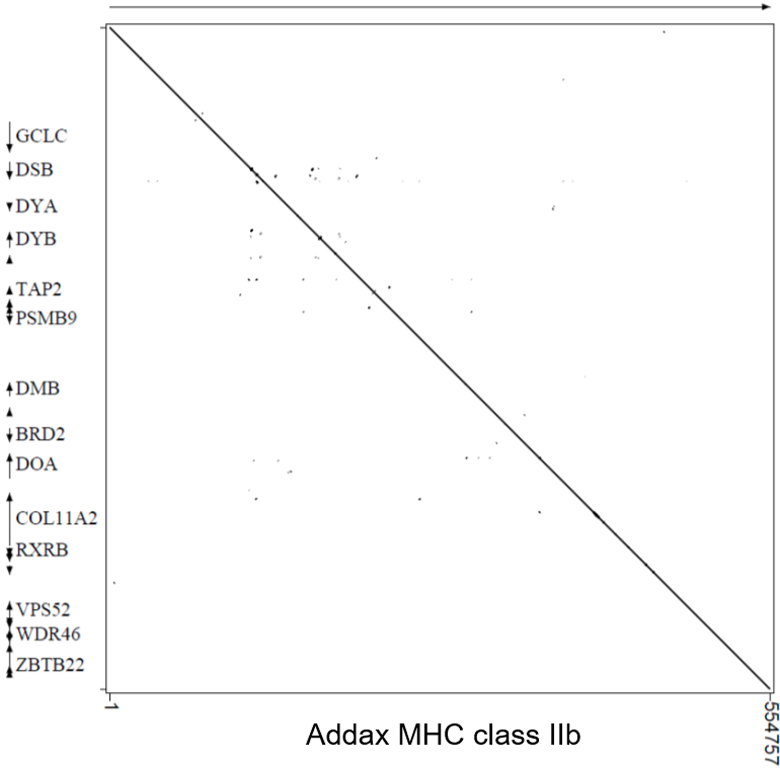


**Figure S4** | **Dot plot analysis of the addax MHC class IIb region.** Genes are listed on the y-axis, with their orientations indicated by arrows.


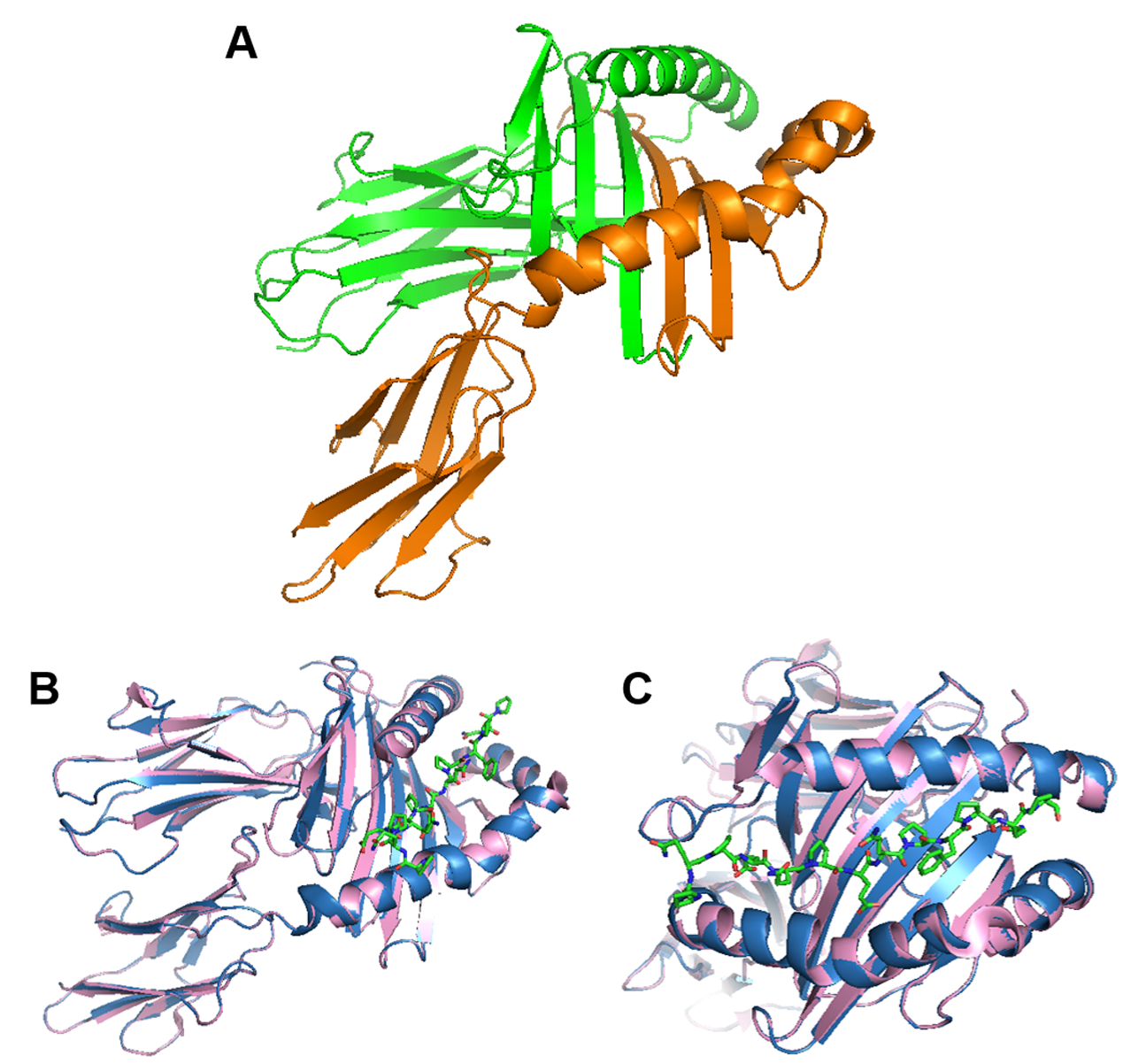


**Figure S5** | **Overall structure of DY in the addax. (A)** The simulated structure of DY in the addax with HLA-DQ2 as a template. The α chain and β chain of DY are colored by green and orange, respectively. **(B-C)** Side and the top views of superimposed addax DY (pink) and HLA-DQ2 (blue). Antigen peptide (from HLA-DQ2) is shown as sticks, with carbon atoms colored green.


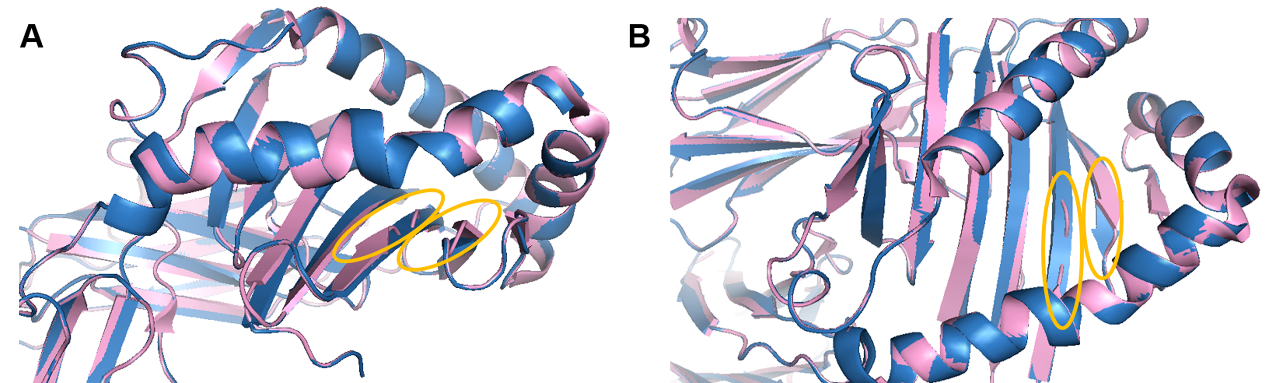


**Figure** **S6** | **Structural differences between DY in the addax and HLA-DQ2.** **(A-B)** Side and the top views of the superimposed structures of addax DY (pink) and HLA-DQ2 (blue). Structure differences between DY in the addax and HLA-DQ2 are indicated by orange circles.


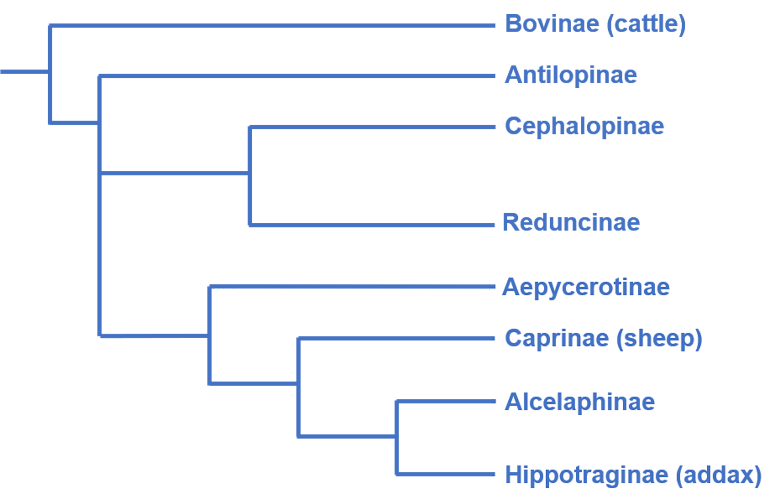


**Figure S7** | Phylogenetic tree of the family Bovidae (adapted from Price *et al* (27)).

**Table S1** | **Constitution of repeat elements (SINEs, LINEs, LTR elements, and DNA elements) in the MHC region of addax, sheep, goat, cattle, water buffalo, sperm whale, pig, horse, and human.**

|  | Addax | Sheep | Goat | Cattle | Water Buffalo | Red Deer | Sperm whale | Pig | Horse | Human |
| --- | --- | --- | --- | --- | --- | --- | --- | --- | --- | --- |
| **SINEs:** | **10.33%** | **10.05%** | **10.21%** | **10.05%** | **10.44%** | **4.76%** | **9.01%** | **18.54%** | **5.17%** | **16.83%** |
|  |  |  |  |  |  |  |  |  |  |  |
| **LINEs:** | **21.41%** | **19.12%** | **21.66%** | **26.99%** | **24.78%** | **7.63%** | **18.94%** | **13.81%** | **27.24%** | **18.27%** |
| LINE1 | 9.81% | 9.03% | 9.93% | 14.13% | 12.18% | 4.30% | 14.65% | 11.01% | 23.09% | 14.74% |
| LINE2 | 2.59% | 2.37% | 2.47% | 2.09% | 2.18% | 1.60% | 3.92% | 2.49% | 3.69% | 3.22% |
| L3/CR1 | 0.19% | 0.19% | 0.17% | 0.16% | 0.18% | 0.11% | 0.30% | 0.25% | 0.32% | 0.21% |
| RTE | 8.82% | 7.53% | 9.09% | 10.61% | 10.25% | 1.61% | 0.07% | 0.06% | 0.14% | 0.00% |
|  |  |  |  |  |  |  |  |  |  |  |
| **LTR elements:** | **4.33%** | **4.37%** | **4.80%** | **4.80%** | **4.58%** | **2.11%** | **4.39%** | **2.82%** | **11.44%** | **11.68%** |
| ERVL | 0.60% | 0.73% | 0.72% | 0.63% | 0.79% | 0.43% | 1.21% | 0.94% | 4.21% | 3.43% |
| ERVL-MaLRs | 0.99% | 0.91% | 0.95% | 0.87% | 0.84% | 0.56% | 1.74% | 0.90% | 2.53% | 2.75% |
| ERV_classI | 1.86% | 1.71% | 1.96% | 2.07% | 1.78% | 0.91% | 1.18% | 0.80% | 4.16% | 3.70% |
| ERV_classII | 0.76% | 0.97% | 1.07% | 1.15% | 1.08% | 0.13% | 0.10% | 0.08% | 0.37% | 1.63% |
|  |  |  |  |  |  |  |  |  |  |  |
| **DNA elements:** | **2.09%** | **2.15%** | **2.09%** | **1.91%** | **2.14%** | **1.21%** | **2.79%** | **2.22%** | **3.52%** | **3.07%** |
|  |  |  |  |  |  |  |  |  |  |  |
| **Total** | **38.16%** | **35.69%** | **38.77%** | **43.75%** | **41.94%** | **15.71%** | **35.13%** | **37.39%** | **47.37%** | **49.85%** |
|  |  |  |  |  |  |  |  |  |  |  |
| LINE/R | 56.10% | 53.57% | 55.88% | 61.69% | 59.10% | 48.56% | 53.91% | 36.94% | 57.50% | 36.65% |
| SINE/R | 27.08% | 28.17% | 26.35% | 22.97% | 24.89% | 30.31% | 25.65% | 49.59% | 10.91% | 33.76% |
| LTR/R | 11.34% | 12.24% | 12.38% | 10.98% | 10.91% | 13.44% | 12.51% | 7.54% | 24.15% | 23.43% |
| RTE/R | 23.11% | 21.10% | 23.46% | 24.24% | 24.44% | 10.27% | 0.19% | 0.16% | 0.30% | 0.00% |

**Table S2** | **GenBank accession numbers of the MHC class II genes of representative mammals used to construct the phylogenetic trees.**

|  | **Sheep** | **Goat** | **Tibetan antelope** | **Cattle** | **Water buffalo** | **White-tailed deer** |
| --- | --- | --- | --- | --- | --- | --- |
| *DMA* | XM_012112128.2 | XM_005696210.3 | XM_005972785.1 | NM_001012674.2 | XM_006050291.2 | XM_020908115.1 |
| *DMB* | XM_012100470.2 | XM_005696209.3 | XM_005972786.1 | NM_001040481.3 | XM_006050290.2 | XM_020908102.1 |
| *DOA* | XM_004018742.3 | XM_013973815.2 | XM_005972796.1 | NM_001205920.1 | XM_006050298.2 | XM_020908113.1 |
| *DOB* | XM_004018738.3 | XM_005696206.3 | XM_005972791.1 | NM_001013600.1 | XM_006050284.2 | XM_020908109.1 |
| *DRA* | XM_012100429.2 | NM_001314188.1 | XM_005957553.1 | NM_001012677.1 | NM_001290945.1 | XM_020916456.1 |
| *DRB* | NM_001280698.1 | XM_018039087.1 | XM_005972792.1 | XM_024984085.1 | XM_025268263.1 | XM_020873369.1 |
| *DQA* | NM_001308597.1 | NM_001285680.2 | XM_005957557.1 | XM_015459849.2 | XM_006047522.2 | XM_020902606.1 |
| *DQB* | XM_027958927.1 | XM_027958925.1 | XM_005957558.1 | NM_001034668.3 | XM_006047521.2 | XM_020908112.1 |
| *DYA* | NM_001123398.2 | XM_005696205.2 | XM_005972799.1 | NM_001012678.1 | XM_025272044.1 | XM_020908117.1 |
| *DYB* | XM_027958632.1 | XM_018039318.1 | XM_005972798.1 | NM_001012679.1 | XM_006050281.2 | XM_020908112.1 |

|  | **Minke whale** | **Sperm whale** | **Bottlenose dolphin** | **Pig** | **Camel** | **Human** |
| --- | --- | --- | --- | --- | --- | --- |
| *DMA* | XM_007186920.1 | XM_024121724.1 | XM_019931419.1 | NM_001004039.1 | XM_010949113.1 | NM_006120.4 |
| *DMB* | XM_007186924.1 | XM_007107873.1 | XM_019931421.1 | NM_001113707.1 | XM_010949114.1 | NM_002118.5 |
| *DOA* | XM_007186913.2 | XM_007107866.2 | NA | NM_001185143.1 | XM_010949105.1 | NM_002119.4 |
| *DOB* | XM_007187065.1 | XM_007107887.2 | XM_019931428.1 | NM_001114064.2 | XM_010949120.1 | NM_002120.4 |
| *DRA* | XM_007193888.2 | XM_007118596.2 | XM_019949740.1 | NM_001113706.1 | XM_010949127.1 | NM_019111.5 |
| *DRB* | XM_028167488.1 | XM_024121957.2 | XM_019949647.1 | NM_001113695.1 | XM_010949121.1 | NM_001243965.1 |
| *DQA* | NA | XM_024121955.2 | XM_019949739.1 | NM_001114062.2 | XM_010949125.1 | NM_002122.3 |
| *DQB* | NA | XM_024121956.2 | XM_019949737.1 | NM_001113694.1 | XM_010949126.1 | NM_001243961.1 |
| *DYA* | NA | NA | NA | NA | NA | NA |
| *DYB* | NA | NA | NA | NA | NA | NA |

**Table S3** | **List of predicted genes in the MHC region of the addax.**

| **Number** | **Description** | **Gene Symbol** | **Start** | **End** | **Strand** | **MHC region** | **Contig** |
| --- | --- | --- | --- | --- | --- | --- | --- |
| 1 | ELOVL fatty acid elongase 5 | ELOVL5 | 81831 | 89406 | - | II a | 1 |
| 2 | SLA class II histocompatibility antigen, DQ haplotype D alpha chain-like | ψDQA3 | 246310 | 249459 | - | II a | 1 |
| 3 | MHC class II OVAR-DR-beta-3, partial | ψDRB3 | 275737 | 297644 | + | II a | 1 |
| 4 | DLA class II histocompatibility antigen, DR-1 beta chain-like | ψDRB1 | 349207 | 353973 | + | II a | 1 |
| 5 | BoLa class II histocompatibility antigen, DQB*0101 beta chain-like | ψDQB2 | 354272 | 356250 | - | II a | 1 |
| 6 | SLA class II histocompatibility antigen, DQ haplotype D alpha chain-like isoform X2 | DQA2 | 378532 | 387854 | + | II a | 1 |
| 7 | MHC class II DQB precursor | DQB1 | 399037 | 411431 | - | II a | 1 |
| 8 | MHC class II DQA, partial | DQA1 | 431426 | 441492 | + | II a | 1 |
| 9 | Major histocompatibility complex, class II, DR beta 4 precursor | DRB4 | 450923 | 458277 | + | II a | 1 |
| 10 | MHC class II antigen | DRB | 471996 | 485917 | + | II a | 1 |
| 11 | BoLA-DRB3 protein | ψDRB3 | 500013 | 510529 | + | II a | 1 |
| 12 | DLA class II histocompatibility antigen, DR-1 beta chain | DRB1 | 529332 | 559156 | + | II a | 1 |
| 13 | Mamu class II histocompatibility antigen, DR alpha chain-like isoform X1 | DRA | 570305 | 578525 | - | II a | 1 |
| 14 | Butyrophilin-like protein 2 | BTNL2 | 594474 | 606644 | + | II a | 1 |
| 15 | Butyrophilin subfamily 1 member A1-like | BT1A1 | 610689 | 618638 | - | II a | 1 |
| 16 | Butyrophilin-like protein 1 | BTNL1 | 621038 | 632675 | + | II a | 1 |
| 17 | Butyrophilin subfamily 1 member A1-like isoform 2 | BT1A1 | 655110 | 685953 | + | II a | 1 |
| 18 | 40S ribosomal protein S2-like | ψRPS2 | 690456 | 691007 | + | II a | 1 |
| 19 | Butyrophilin subfamily 1 member A1 | BTNL1 | 823286 | 832975 | + | II a | 1 |
| 20 | Uncharacterized protein C6orf10-like | TSBP1 | 904766 | 937584 | + | II a | 1 |
| 21 | Butyrophilin subfamily 1 member A1-like | BTNL1 | 1026831 | 1040436 | - | II a | 1 |
| 22 | Butyrophilin subfamily 3 member A3-like | BTNL3 | 1045292 | 1063459 | - | II a | 1 |
| 23 | Neurogenic locus notch homolog protein 4 isoform X2 | NOTCH4 | 1085591 | 1110054 | + | III | 1 |
| 24 | Pre-B-cell leukemia transcription factor 2 | PBX2 | 1114684 | 1117956 | + | III | 1 |
| 25 | Advanced glycosylation end-product specific receptor | AGER | 1119668 | 1123388 | + | III | 1 |
| 26 | E3 ubiquitin-protein ligase RNF5 | RNF5 | 1123988 | 1124910 | - | III | 1 |
| 27 | 1-acyl-sn-glycerol-3-phosphate acyltransferase alpha precursor | AGPAT1 | 1132666 | 1135792 | + | III | 1 |
| 28 | Epidermal growth factor-like protein 8 | EGFL8 | 1136230 | 1137923 | - | III | 1 |
| 29 | Lysosomal thioesterase PPT2 isoform X1 | PPT2 | 1139347 | 1147374 | - | III | 1 |
| 30 | Proline-rich transmembrane protein 1 isoform X1 | PRRT1 | 1148175 | 1161628 | + | III | 1 |
| 31 | 506-binding protein-like isoform X1 | FKBPL | 1166125 | 1167162 | + | III | 1 |
| 32 | Cyclic AMP-dependent transcription factor ATF-6 beta isoform X2 | ATF6B | 1167719 | 1177124 | + | III | 1 |
| 33 | Tenascin XB | TNXB | 1179119 | 1240351 | + | III | 1 |
| 34 | Complement C4-A-like | C4A | 1240852 | 1248682 | - | III | 1 |
| 35 | Steroid 21-hydroxylase-like | CYP21A | 1262659 | 1265556 | - | III | 1 |
| 36 | Complement C4-like protein | C4 | 1269091 | 1282056 | - | III | 1 |
| 37 | Helicase SKI2W | SKIV2L | 1283283 | 1297529 | - | III | 1 |
| 38 | Negative elongation factor E isoform 1 | NELFE | 1298253 | 1303379 | + | III | 1 |
| 39 | Complement factor B | CFB | 1303651 | 1309448 | - | III | 1 |
| 40 | Complement C2 isoform X1 | C2 | 1310297 | 1321668 | - | III | 1 |
| 41 | Zinc finger and BTB domain-containing protein 12 | ZBTB12 | 1351475 | 1352854 | + | III | 1 |
| 42 | Histone-lysine N-methyltransferase EHMT2 | EHMT2 | 1355052 | 1367844 | + | III | 1 |
| 43 | Choline transporter-like protein 4 isoform 1 | SLC44A2 | 1368670 | 1381752 | + | III | 1 |
| 44 | Sialidase-1 | NEU1 | 1382852 | 1385612 | + | III | 1 |
| 45 | Heat shock 70 kDa protein 1-like | HSPA1L | 1402612 | 1417721 | - | III | 1 |
| 46 | Heat shock 70 kDa protein 1B | HS71B | 1420991 | 1422916 | - | III | 1 |
| 47 | Heat shock 70 kDa protein 1-like | HSPA1L | 1424922 | 1426847 | + | III | 1 |
| 48 | LSM2 homolog, U6 small nuclear RNA and mRNA degradation associated | LSM2 | 1428283 | 1433185 | + | III | 1 |
| 49 | Valyl-tRNA synthetase | VARS | 1434450 | 1445624 | + | III | 1 |
| 50 | Von Willebrand factor A domain-containing protein 7 | VWA7 | 1446430 | 1454513 | + | III | 1 |
| 51 | Suppressor APC domain-containing protein 1 | SAPCD1 | 1455422 | 1456872 | - | III | 1 |
| 52 | MutS protein homolog 5 | MSH5 | 1457394 | 1470645 | - | III | 1 |
| 53 | Chloride intracellular channel protein 1, partial | CLIC1 | 1474779 | 1479685 | + | III | 1 |
| 54 | N(G),N(G)-dimethylarginine dimethylaminohydrolase 2 | DDAH2 | 1481364 | 1483592 | + | III | 1 |
| 55 | Protein G6b isoform X3 | MPIG6B | 1485610 | 1487276 | - | III | 1 |
| 56 | Lymphocyte antigen 6 complex locus protein G6c | LY6G6C | 1488580 | 1490140 | + | III | 1 |
| 57 | Lymphocyte antigen 6 complex locus protein G6d | LY6G6D | 1491993 | 1493739 | - | III | 1 |
| 58 | Sperm acrosome membrane-associated protein 4-like isoform X1 | LY6G6E | 1495727 | 1496948 | + | III | 1 |
| 59 | Lymphocyte antigen 6 complex locus protein G6f precursor | LY6G6F | 1499010 | 1501629 | - | III | 1 |
| 60 | Abhydrolase domain-containing protein 16A | ABHD16A | 1503947 | 1516193 | + | III | 1 |
| 61 | Lymphocyte antigen 6 complex locus protein G5c | LY6G5C | 1521052 | 1523404 | + | III | 1 |
| 62 | Lymphocyte antigen 6 complex locus protein G5b isoform X2 | LY6G5B | 1526198 | 1527210 | - | III | 1 |
| 63 | Casein kinase II subunit beta isoform X1 | CSNK2B | 1528172 | 1531092 | - | III | 1 |
| 64 | G patch domain and ankyrin repeat-containing protein 1 isoform X1 | GPANK1 | 1531650 | 1535018 | + | III | 1 |
| 65 | Uncharacterized protein C6orf47 homolog | C20H6orf47 | 1537287 | 1538180 | + | III | 1 |
| 66 | Apolipoprotein M isoform X2 | APOM | 1539167 | 1541515 | - | III | 1 |
| 67 | Large proline-rich protein BAG6 isoform X1 | BAG6 | 1542829 | 1554535 | + | III | 1 |
| 68 | Protein PRRC2A | PRRC2A | 1555724 | 1568633 | - | III | 1 |
| 69 | Allograft inflammatory factor 1-like protein | AIF1 | 1582853 | 1587716 | - | III | 1 |
| 70 | Natural cytotoxicity triggering receptor 3 | NCR3 | 1600771 | 1604957 | + | III | 1 |
| 71 | Leukocyte-specific transcript 1 protein | LST1 | 1605704 | 1607990 | - | III | 1 |
| 72 | Lymphotoxin-beta | LTB | 1610366 | 1612905 | + | III | 1 |
| 73 | Tumor necrosis factor isoform X1 | TNF | 1615884 | 1618021 | - | III | 1 |
| 74 | Lymphotoxin-alpha | LTA | 1619721 | 1620633 | - | III | 1 |
| 75 | NF-kappa-B inhibitor-like protein 1 | NFKBIL1 | 1632263 | 1632973 | - | III | 1 |
| 76 | V-type proton ATPase subunit G 2 isoform X1 | ATP6V1G2 | 1642799 | 1643959 | + | III | 1 |
| 77 | Spliceosome RNA helicase DDX39B isoform X2 | DDX39B | 1648264 | 1657694 | + | I | 1 |
| 78 | Mitochondrial coiled-coil domain protein 1 | MCCD1 | 1658172 | 1659266 | - | I | 1 |
| 79 | BOLA class I histocompatibility antigen, alpha chain BL3-7-like | MHC-I | 1672126 | 1685351 | + | I | 1 |
| 80 | Interferon-induced transmembrane protein 3-like | ψIFIM3 | 1699610 | 1699831 | + | I | 1 |
| 81 | MHC class I heavy chain | MHC-I | 1745648 | 1750060 | + | I | 1 |
| 82 | MHC class I heavy chain | MHC-I | 1772173 | 1780177 | + | I | 1 |
| 83 | MHC class I polypeptide-related sequence B-like, partial | MICB | 1784105 | 1788601 | - | I | 1 |
| 84 | OCT4 protein | POU5F | 1813069 | 1817544 | + | I | 1 |
| 85 | Transcription factor 19 | TCF19 | 1819374 | 1821701 | - | I | 1 |
| 86 | Coiled-coil alpha-helical rod protein 1 | CCHCR1 | 1822475 | 1834499 | + | I | 1 |
| 87 | Psoriasis susceptibility 1 candidate gene 2 protein homolog | PSORS1C2 | 1836684 | 1837733 | + | I | 1 |
| 88 | Corneodesmosin precursor | CDSN | 1840265 | 1856812 | + | I | 1 |
| 89 | Uncharacterized protein C6orf15 homolog isoform X1 | C20Horf15 | 1860736 | 1861855 | + | I | 1 |
| 90 | MHC class I alpha chain precursor, partial | MHC1 | 1888514 | 1891409 | + | I | 1 |
| 91 | Diffuse panbronchiolitis critical region | DPCR1 | 1951439 | 1955687 | - | I | 1 |
| 92 | Surfactant-associated protein 2 | SFTA2 | 1969458 | 1971714 | + | I | 1 |
| 93 | General transcription factor IIH subunit 4 | GTF2H4 | 1986076 | 1993342 | - | I | 1 |
| 94 | Epithelial discoidin domain-containing receptor 1 | DDR1 | 2001173 | 2011084 | - | I | 1 |
| 95 | Immediate early response 3 | IER3 | 2110489 | 2111818 | + | I | 1 |
| 96 | Flotillin-1 | FLOT1 | 2112745 | 2122574 | + | I | 1 |
| 97 | Tubulin, beta 5 | TUBB | 2124672 | 2127919 | - | I | 1 |
| 98 | Mediator of DNA damage checkpoint protein 1 isoform X2 | MDC1 | 2128812 | 2143874 | + | I | 1 |
| 99 | Nurim | NRM | 2147180 | 2150060 | + | I | 1 |
| 100 | Phostensin | PPP1R18 | 2151556 | 2160096 | + | I | 1 |
| 101 | Putative pre-mRNA-splicing factor ATP-dependent RNA helicase DHX16 | DHX16 | 2163505 | 2174201 | + | I | 1 |
| 102 | Uncharacterized protein C6orf136 | C6orf136 | 2174467 | 2178416 | - | I | 1 |
| 103 | Alpha-tubulin N-acetyltransferase 1 | ATAT1 | 2190066 | 2202050 | - | I | 1 |
| 104 | Serine/threonine-protein phosphatase 1 regulatory subunit 10 isoform X2 | PPP1R10 | 2205334 | 2213756 | + | I | 1 |
| 105 | ATP-binding cassette sub-family F member 1 isoform X4 | ABCF1 | 2223013 | 2235242 | - | I | 1 |
| 106 | Proline-rich protein 3 | PRR3 | 2243493 | 2247279 | - | I | 1 |
| 107 | Guanine nucleotide-binding protein-like 1 | GNL1 | 2248437 | 2255830 | + | I | 1 |
| 108 | Ribonuclease P protein subunit p21 isoform X4 | RPP21 | 2266494 | 2283632 | - | I | 1 |
| 109 | Tripartite motif-containing protein 39 | TRIM39 | 2285634 | 2304127 | - | I | 1 |
| 110 | BOLA class I histocompatibility antigen, alpha chain BL3-7-like isoform X1 | MHC-I | 2318399 | 2325701 | + | I | 1 |
| 111 | BOLA class I histocompatibility antigen, alpha chain BL3-7-like | MHC-I | 2349081 | 2351466 | - | I | 1 |
| 112 | MHC class I antigen | MHC-I | 2407154 | 2417126 | - | I | 1 |
| 113 | Tripartite motif-containing protein 26 | TRIM26 | 2468930 | 2477473 | + | I | 1 |
| 114 | Tripartite motif-containing protein 15 | TRIM15 | 2494882 | 2500372 | - | I | 1 |
| 115 | Tripartite motif-containing protein 10 | TRIM10 | 2503349 | 2511083 | + | I | 1 |
| 116 | Tripartite motif-containing protein 40 isoform X1 | TRIM40 | 2512687 | 2526787 | - | I | 1 |
| 117 | E3 ubiquitin-protein ligase TRIM31 | TRIM31 | 2535563 | 2565444 | + | I | 1 |
| 118 | RING finger protein 39, partial | RNF39 | 2587733 | 2592654 | + | I | 1 |
| 119 | Protein phosphatase 1 regulatory subunit 11, partial | PPP1R11 | 2594512 | 2605597 | - | I | 1 |
| 120 | Putative uncharacterized protein ZNRD1-AS1-like | ZNRD1 | 2607012 | 2607263 | + | I | 1 |
| 121 | Zinc finger protein 57 homolog | ZFP57 | 2613198 | 2625343 | + | I | 1 |
| 122 | Myelin-oligodendrocyte glycoprotein isoform X7 | MOG | 2626311 | 2646961 | - | I | 1 |
| 123 | Gamma-aminobutyric acid type B receptor subunit 1 | GABBR1 | 2656959 | 2668204 | + | I | 1 |
| 124 | Glutamate--cysteine ligase catalytic subunit | GCLC | 79269 | 104585 | + | II b | 2 |
| 125 | MHC class II antigen DS beta | DSB | 113207 | 127454 | + | II b | 2 |
| 126 | MHC class II antigen DY alpha precursor | DYA | 147743 | 153550 | + | II b | 2 |
| 127 | Major histocompatibility complex, class II, DY beta precursor | DYB | 171540 | 184562 | - | II b | 2 |
| 128 | HLA class II histocompatibility antigen, DO beta chain-like | DOB | 191275 | 193702 | - | II b | 2 |
| 129 | Antigen peptide transporter 2 | TAP2 | 217046 | 223902 | - | II b | 2 |
| 130 | Proteasome subunit beta type-8 | PSMB8 | 228029 | 231663 | - | II b | 2 |
| 131 | Antigen peptide transporter 1 | TAP1 | 233225 | 241484 | - | II b | 2 |
| 132 | Proteasome subunit beta type-9 | PSMB9 | 242081 | 248507 | + | II b | 2 |
| 133 | HLA class II histocompatibility antigen, DM beta chain-like | DMB | 297817 | 309290 | - | II b | 2 |
| 134 | HLA class II histocompatibility antigen, DM alpha chain-like | DMA | 319432 | 324063 | - | II b | 2 |
| 135 | Bromodomain-containing protein 2 isoform X1 | BRD2 | 336658 | 347642 | + | II b | 2 |
| 136 | HLA class II histocompatibility antigen, DO alpha chain-like isoform X1 | DOA | 357162 | 377852 | - | II b | 2 |
| 137 | Collagen alpha-2(XI) chain | COL11A2 | 390448 | 434244 | - | II b | 2 |
| 138 | Retinoid X receptor, beta | RXRB | 436681 | 441974 | - | II b | 2 |
| 139 | Zinc transporter SLC39A7 | SLC39A7 | 442963 | 445996 | + | II b | 2 |
| 140 | Estradiol 17-beta-dehydrogenase 8 isoform X1 | HSD17B8 | 446806 | 447989 | + | II b | 2 |
| 141 | E3 ubiquitin-protein ligase RING1 | RING1 | 451684 | 458517 | + | II b | 2 |
| 142 | SAC2 suppressor of actin mutations 2-like protein | VPS52 | 481498 | 496546 | - | II b | 2 |
| 143 | 40S ribosomal protein S18 isoform 1 | RPS18 | 496972 | 501754 | + | II b | 2 |
| 144 | Beta-1,3-galactosyltransferase 4 isoform 1 | B3GALT4 | 502815 | 503951 | + | II b | 2 |
| 145 | WD repeat-containing protein 46 isoform 1 | WDR46 | 504656 | 513995 | - | II b | 2 |
| 146 | Prefoldin subunit 6 | PFDN6 | 514655 | 515588 | + | II b | 2 |
| 147 | Ral guanine nucleotide dissociation stimulator-like 2 | RGL2 | 516907 | 520136 | - | II b | 2 |
| 148 | TAP binding protein | TAPBP | 523382 | 533905 | - | II b | 2 |
| 149 | Zinc finger and BTB domain-containing protein 22 | ZBTB22 | 534893 | 536803 | - | II b | 2 |
| 150 | Death domain-associated protein 6 isoform X1 | DAXX | 538416 | 542617 | - | II b | 2 |

**Table S4** | **List of predicted tRNA genes in the MHC region of the addax.**

| **No. of tRNA** | **tRNA Begin** | **Bounds End** | **tRNA Type** | **Anti Codon** | **Contig** |
| --- | --- | --- | --- | --- | --- |
| 1 | 12128 | 12200 | Glu | TTC | 1 |
| 2 | 198055 | 198126 | SeC | TCA | 1 |
| 3 | 282813 | 282885 | Arg | GCG | 1 |
| 4 | 467658 | 467730 | Arg | GCG | 1 |
| 5 | 583329 | 583403 | Ile | TAT | 1 |
| 6 | 613396 | 613468 | Tyr | ATA | 1 |
| 7 | 777898 | 777970 | Cys | ACA | 1 |
| 8 | 885775 | 885847 | Cys | GCA | 1 |
| 9 | 961886 | 961956 | Cys | GCA | 1 |
| 10 | 1373325 | 1373397 | Gly | TCC | 1 |
| 11 | 1401041 | 1401113 | Lys | CTT | 1 |
| 12 | 1471948 | 1472020 | Gly | GCC | 1 |
| 13 | 1501943 | 1502013 | Leu | CAA | 1 |
| 14 | 1598250 | 1598321 | Gly | CCC | 1 |
| 15 | 1669367 | 1669440 | Glu | TTC | 1 |
| 16 | 1691851 | 1691923 | SeC | TCA | 1 |
| 17 | 1796794 | 1796866 | Tyr | GTA | 1 |
| 18 | 1800528 | 1800600 | Cys | GCA | 1 |
| 19 | 1835329 | 1835401 | Gly | CCC | 1 |
| 20 | 1932932 | 1933004 | Glu | TTC | 1 |
| 21 | 2038164 | 2038235 | Gly | GCC | 1 |
| 22 | 2297844 | 2297915 | Cys | GCA | 1 |
| 23 | 2386019 | 2386091 | Gly | GCC | 1 |
| 24 | 2407333 | 2407405 | Cys | GCA | 1 |
| 25 | 2557350 | 2557425 | Cys | ACA | 1 |
| 26 | 2614972 | 2615044 | Glu | CTC | 1 |
| 27 | 2649766 | 2649836 | SeC | TCA | 1 |
| 28 | 2504589 | 2504517 | Gly | TCC | 1 |
| 29 | 2160772 | 2160701 | Glu | TTC | 1 |
| 30 | 2038011 | 2037939 | Cys | GCA | 1 |
| 31 | 1972281 | 1972209 | Cys | GCA | 1 |
| 32 | 1971511 | 1971441 | Trp | CCA | 1 |
| 33 | 1866876 | 1866803 | Glu | TTC | 1 |
| 34 | 1848480 | 1848407 | Trp | CCA | 1 |
| 35 | 1684685 | 1684613 | Ser | GCT | 1 |
| 36 | 1588526 | 1588454 | Trp | CCA | 1 |
| 37 | 1415875 | 1415803 | Gly | GCC | 1 |
| 38 | 1358477 | 1358405 | Gly | CCC | 1 |
| 39 | 1338579 | 1338507 | Gly | ACC | 1 |
| 40 | 1267744 | 1267672 | Gly | ACC | 1 |
| 41 | 1103872 | 1103800 | Trp | CCA | 1 |
| 42 | 813287 | 813216 | Trp | CCA | 1 |
| 43 | 602973 | 602901 | Cys | GCA | 1 |
| 44 | 212863 | 212792 | Cys | ACA | 1 |
| 45 | 3764 | 3694 | Trp | CCA | 1 |
| 46 | 377247 | 377318 | SeC | TCA | 2 |
| 47 | 462994 | 463066 | SeC | TCA | 2 |
| 48 | 483498 | 483570 | Arg | CCT | 2 |
| 49 | 326610 | 326538 | Gly | TCC | 2 |
| 50 | 65352 | 65282 | SeC | TCA | 2 |
